# Exposure of Agriculture Workers to Pesticides: The Effect of Heat on Protective Glove Performance and Skin Exposure to Dichlorvos

**DOI:** 10.3390/ijerph16234798

**Published:** 2019-11-29

**Authors:** Leigh Thredgold, Sharyn Gaskin, Chloe Quy, Dino Pisaniello

**Affiliations:** 1 Adelaide Exposure Science and Health, School of Public Health, University of Adelaide, Adelaide, South Australia 5000, Australia; 2 AgroParisTech, Paris Institute of Technology for Life, Food and Environmental Sciences, 75231 Paris, France

**Keywords:** organophosphate, protective gloves, dermal, percutaneous penetration

## Abstract

Dichlorvos is a toxic organophosphate insecticide that is used in agriculture and other insecticide applications. Dermal uptake is a known exposure route for dichlorvos and chemical protective gloves are commonly utilized. Chemical handling and application may occur in a variety of thermal environments, and the rates of both chemical permeation through gloves and transdermal penetration may vary significantly with temperature. There has been no published research on the temperature-dependent kinetics of these processes for dichlorvos and thus, this study reports on the effects of hot conditions for the concentrated and application strength chemical. Dichlorvos breakthrough times for non-disposable polyvinyl chloride (PVC) gloves at 60 °C were approximately halved compared to 25 °C for the concentrate (2 vs. 4 h) and more than halved at application strength (3 vs. >8 h). From permeation experiments covering 15–60 °C, there was a 460-fold increase in cumulative permeation over 8 h for the concentrated dichlorvos and the estimated activation energy halved. Elevated temperature was also shown to be a significant factor for human skin penetration increasing the cumulative penetration of concentrate dichlorvos from 179 ± 37 to 1315 ± 362 µg/cm^2^ (*p* = 0.0032) and application strength from 29.8 ± 5.7 to 115 ± 19 µg/cm^2^ (*p* = 0.0131). This work illustrates the important role temperature plays in glove performance and health risk via dermal exposure. As such, it is important to consider in-use conditions of temperature when implementing chemical hygiene programs.

## 1. Introduction

Dichlorvos (2,2-dichlorovinyl dimethyl phosphate) is an organophosphate pesticide used in the agriculture industry to control a wide variety of insects in greenhouses and outdoor fruit and vegetable crops. The primary mode of action is acetylcholinesterase inhibition which causes over stimulation and subsequent prevention of nerve transmission. Exposure to dichlorvos can lead to a number of potential acute or chronic health effects ranging from perspiration and vomiting to fatigue and headache, whilst exposure to elevated concentrations can also result in more serious health concerns such as convulsions and coma [1]. In the developed world, the use of dichlorvos has been heavily regulated due its high toxicity and worker exposure potential. For example, in Australia and the USA its use is heavily regulated and restricted to licensed users for the professional agricultural, pest control, or veterinary sectors as a disinfectant fumigant whereas in many European Union countries dichlorvos products are completely banned [2,3]. In developing countries, dichlorvos along with other organophosphate pesticides (OPs) are largely unregulated and are still widely available and utilized in the agriculture and horticulture sectors [3]. Owing to its continued use, there is high potential for occupational exposure and as a result, OPs such as dichlorvos are associated with significant morbidity and mortality in these countries [4].

The primary exposure routes for dichlorvos in occupational settings are the respiratory and dermal pathways [5]. Respiratory inhalation arises via the presence of dichlorvos vapor and/or airborne aerosol from spray application and is of higher risk in the absence of respiratory protection or in poorly ventilated environments. In agricultural settings, despite spray application being common, it is reported that only 10% of total pesticide exposure occurs via the respiratory route [6]. The remaining 90% is attributed to dermal absorption or ingestion. Dermal absorption occurs through direct skin contact (splash or aerosol) or indirect contact with contaminated tools or clothing. The American Conference of Governmental Industrial Hygienists (ACGIH) threshold limit values (TLV) for dichlorvos has a “skin notation” which is assigned to chemicals where significant systemic toxicity may occur via the dermal route and/or to warn of significant dermal absorption potential. Thus, dermal exposure to dichlorvos warrants particular attention.

Personal protective equipment (PPE) provides front line protection against chemical exposure, particularly in agricultural settings involving outdoor environments. MacFarlane et al. [7] outlined and evaluated the literature concerning the role of PPE with occupational end use of pesticides. Other studies have also evaluated the performance of PPE in relation to dermal protection from a wide range of pesticides [8,9,10,11]. For dichlorvos specifically, only protection performance experiments using rubberized fabric (thickness: 0.42 mm) [12] and ‘every-day’ clothing [13] have been reported. No studies have been found evaluating the performance of recommended gloves for protection against skin exposure to dichlorvos under realistic in-use workplace conditions.

However, a limited number of studies have specifically investigated the skin penetration potential of dichlorvos in humans. Gold et al. [14] performed an in vivo study using periodic biological monitoring of blood and urine to assess dermal penetration in dichlorvos applicators (0.5% strength) who wore respiratory protection. They also utilized pads attached to outer clothing and skin beneath clothing to assess the dermal exposure potential. Their findings of “no significant risk”, in terms of acute toxicity to applicators, were in contrast to Perger et al. (in German) [15] who assessed total body exposure using skin pads and monitored serum cholinesterase activity in workers applying concentrated dichlorvos (45%) with and without respiratory protection and rubber gloves. More recently, Moore et al. [16] used in vitro methodology to investigate the skin permeation of organophosphates including dichlorvos in different vehicles. A number of animal-based studies have also documented the skin permeability of dichlorvos [17,18,19,20].

Despite studies having demonstrated the ability for dichlorvos to penetrate human skin, there remains a distinct lack of data surrounding the performance of chemical protective gloves under varying environmental conditions encountered in real-life occupational scenarios, and the resulting dose to the skin based on that performance. The selection of protective gloves is typically based on general glove selection guides by manufacturers and/or safety data sheets that are informed by glove permeation tests under restricted conditions e.g., at room temperature. In agricultural settings where significant seasonal temperature fluctuations are commonly experienced, this may influence the performance of the protective material. Pharmaceutical studies have shown the application of heat increases transdermal penetration as a result of increased diffusion and intercellular mass flow [21,22,23]. A comparable effect on chemical protective gloves might occur due to increased diffusion and solubility. Such combined effects in gloves and skin may increase uptake. Overall, there remains a significant knowledge gap surrounding the role of environmental conditions on dermal exposure risks.

In this paper, the effect of heat on the performance of chemical protective gloves and subsequent dermal exposure potential to dichlorvos is explored in the context of concentrations and timeframes of occupational relevance for the agriculture industry. We report experimental data from standardized testing protocols and use this to determine the effect of heat on (1) the performance of recommended chemical protective gloves and (2) transdermal penetration of dichlorvos. The data generated address an existing knowledge gap around the effectiveness of chemical protective gloves as a defense against chemical exposure during handling under elevated temperature conditions, and gives further insight into the effect of temperature on transdermal penetration of dichlorvos. Outcomes may help to inform recommendations regarding protective glove use under elevated temperature conditions.

## 2. Materials and Methods

### 2.1. Overall Approach

The experiments were designed to assess two situations—use of concentrate (undiluted; 1398 g/L) and application strength (6 g/L) dichlorvos. These concentrations are physiologically relevant to the occupational exposure scenario of a farm worker mixing a concentrate solution with water to produce the most commonly used (according to product label) solution for insecticide spraying. Product labels and safety data sheets (SDS) for formulated agricultural dichlorvos products were consulted to determine the type of chemical protective gloves recommended for dermal protection during handling [24]. As a result, elbow length polyvinyl chloride (PVC) gloves (PVC45, ProChoice, Melbourne, Australia) were used for glove performance experiments in this study.

### 2.2. Materials

Dichlorvos (technical grade, 98.9%) was supplied by Accensi Pty Ltd. (Brisbane, Australia). Analytical standards for analysis were prepared from Dichlorvos PESTANAL^TM^ analytical standard (>98%) supplied by Sigma Aldrich (Sydney, Australia). HPLC solvents used were acetonitrile (>99.5%, Sigma Aldrich, Sydney, Australia) and purified water (IBIS Plus 6 lph, IBIS Technology, Perth, Australia).

### 2.3. Glove Performance Tests

Glove performance (permeation resistance) tests were conducted using American Society for Testing and Materials (ASTM) permeation test cells according to the Australian/New Zealand Standard^TM^ for occupational protective gloves (AS/NZS 2161.10.3:2005). Swatches (4.5 cm in diameter; 15.9 cm^2^ surface area) were cut from the palm area of the gloves and thickness measurements performed using a digital thickness gauge (547–301, 0.01–10 mm, Mitutoyo) at five different points (mean thickness range 1.05–1.26 mm). A two-compartment cell (ASTM permeation test cell) was used to sandwich glove swatches between a donor chamber and a receptor chamber. A diffusion-available surface area of 5.31 cm^2^ was exposed to the donor chamber and the volume of the corresponding receptor chamber was 16.4 mL water was used as receptor fluid and was continuously stirred. Samples (200 µL) were taken at regular intervals from the receptor chamber to determine dichlorvos permeation and subsequently replaced with fresh receptor fluid.

### 2.4. Skin Permeation Tests

Complementary skin penetration studies for dichlorvos were performed in vitro using static Franz diffusion cells (0.64 cm^2^ diffusion area and 5 mL receptor volume, PermeGear, Hellertown, PA, USA) with physiological saline (0.9% w/v NaCl) receptor fluid according to OECD guidelines [25]. The epidermal layer was harvested from full thickness human abdominal skin via heat separation, donated after cosmetic reduction surgery with donor consent (ethics approval #273.10 SACHRE), within one hour of excision. Electrical impedance was utilized to confirm skin barrier integrity in vitro prior to exposure [26,27,28,29]. Infinite dose experiments were performed to permit calculation of steady-state fluxes and permeability coefficients. This was achieved by placing 200 µL of liquid dichlorvos as application strength (1.9 mg/cm^2^) or concentrate (437 mg/cm^2^) directly to the surface of the skin. An exposure duration of 8 h was chosen to represent a work exposure scenario, and receptor fluid was sampled at 0, 0.5, 1, 2, 3, 4, 6, and 8 h. Receptor fluid samples were 200 µL and were subsequently replenished with fresh receptor fluid. Studies were performed on a minimum of 4 skin replicates for each concentration using epidermis from three donors (female; 24–57 years of age).

### 2.5. Temperature Studies

To investigate the effect of temperature on glove performance and skin permeation, experiments were performed across a range of temperatures. Glove performance studies were conducted at 15, 25, 35, 40, 45, and 60 °C in order to reflect the wide ranging temperatures experienced in real world agricultural settings. A minimum of 3 replicates per temperature were performed and gloves were exposed to concentrate and application strength dichlorvos for up to 8 h to represent a worker exposure scenario. Skin permeation experiments were conducted only at 25 and 37 °C to evaluate the fundamental effect of temperature on skin permeation. Temperature studies were conducted by placing the respective experimental setup in a temperature controlled incubator (ICCBOD-140, LABEC, Sydney, Australia) that maintained the temperature at the required level (±0.2 °C throughout the 8 h experimental timeframe). For each test concentration and temperature, experiments were repeated up to five times to ensure the reproducibility and reliability of the data.

### 2.6. Dichlorvos Quantification

Quantification of dichlorvos in receptor fluid samples was achieved using high performance liquid chromatography with ultraviolet/visible detection (HPLC-UV). Analysis was performed using a Perkin-Elmer Flexar^TM^ HPLC system comprising of solvent manager, isocratic LC pump, and UV absorbance detector controlled by Perkin Elmer TotalChrom (v6.2.0.0.1) software (Perkin Elmer, Melbourne, Australia). The system was run using a 40:60 acetonitrile: water mobile phase with detection at a UV absorbance wavelength of 220 nm. Samples were manually injected (20 µL) followed by separation on a Phenomonex Kinetex^®^ C18 column (150 × 4.6 mm 5 µm). The achieved limit of detection (LOD) was 0.14 µg/mL.

### 2.7. Data Treatment and Statistics

The concentration of dichlorvos in the receptor fluid for both skin and glove permeation studies was corrected for previous sample removal and the resultant cumulative amount per unit surface area (µg/cm^2^) plotted against time. The linear portion of the plots for skin permeation were used to determine estimates of the steady state flux (*J_ss_*) and the permeability coefficient (*K_p_*) was then calculated as:

(1) $$K_{p}\;=\;\frac{J_{ss}}{C_{v}},$$

where *J_ss_* is the steady state flux and *C_v_* is the concentration of dichlorvos in the donor solution. The cumulative permeation amount from the glove studies was then used to calculate the corresponding dichlorvos permeation rate (µg/cm^2^/min). The permeation rate was calculated using the following formula outlined in AS/NZS standard 2161.10.3 (2005):

(2) $$P_{i}\;=\;\frac{\left[\left(C_{i}-C_{i-l}\right)\left[\frac{V_{t}-\;V_{s}}{V_{t}}\right]V_{t}\right]}{\left(t_{i}-\;t_{i-l}\right)A},$$

where *P_i_* is the permeation rate (µg/cm^2^/min), *A* is the area of material specimen in contact (cm^2^), *i* is an indexing number assigned to each discrete sample, starting with *i* = 1 for the first sample, *t_i_* is the time at which discrete sample *i* was removed (mins), *C_i_* is the concentration of dichlorvos in receptor fluid at time *t_i_* (µg/L), *V_t_* is the total volume of the receptor fluid (L), and *V_s_* is the volume of discrete sample removed from the receptor fluid (L). Breakthrough time is reported according to the AS/NZS standard 2161.10.3 [30] which defines breakthrough as occurring when the permeation rate reaches 1 µg/cm^2^/min.

Activation energy (*E_a_*) provides a measure of the resistance to diffusion of a penetrant to cross a given membrane and has been previously used as an empirical measure of the influence of heat on membrane diffusivity [23]. Using the glove permeation data, the activation energy (*E_a_*) for dichlorvos concentrate was calculated across different experimental temperature ranges, in accordance with the Arrhenius equation as used by Akomeah et al. [23]:

(3) $$logK_{p}\;=\;\frac{logA-\;E_{a}}{2.303RT},$$

where *T* is the absolute temperature (K), *A* is the frequency factor, and *R* is the molar gas constant. A plot of *logK_p_* against 1/T was used to calculate *E_a_* over different temperature ranges.

Statistical comparisons of glove permeation outcomes under various exposure conditions (e.g., temperature) were performed using analysis of variance, and Tukey post hoc multiple comparisons tests. Independent samples *t*-test allowed for comparison between skin permeation outcomes at a given exposure time point (8 h). Statistical analyses were performed using GraphPad Prism v.7 software (GraphPad, San Diego, CA, USA).

## 3. Results

### 3.1. Glove Performance Studies

**Figure 1:**
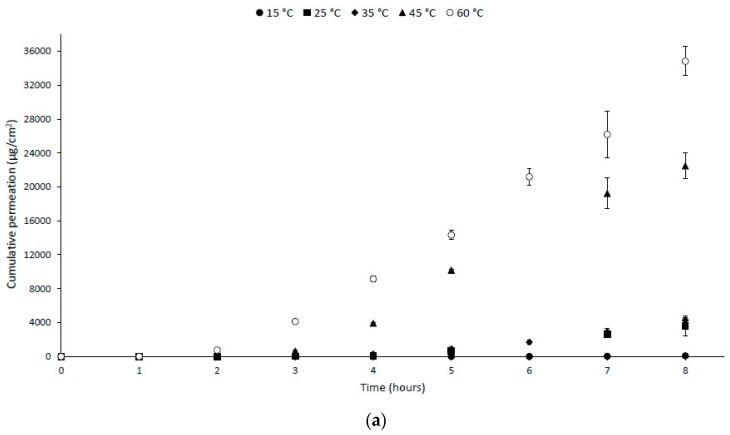

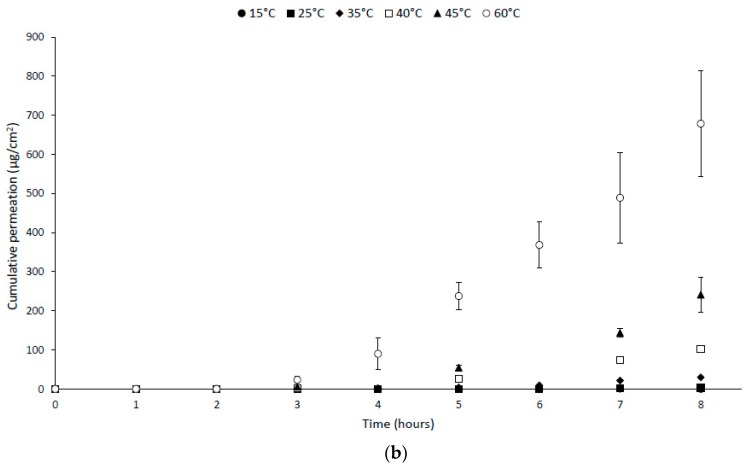
Cumulative permeation of (**a**) concentrate (1398 g/L) and (**b**) application strength (6 g/L) dichlorvos through PVC gloves over 8 h for temperatures ranging from 15 to 60 °C. Values are presented as mean ± SEM (n ≥ 3).

Figure 1 displays dichlorvos permeation curves arising from PVC glove exposure to concentrate (1398 g/L) and application strength (6 g/L) dichlorvos at temperatures ranging from 15 to 60 °C. Cumulative permeation through PVC gloves resulting from exposure to concentrate dichlorvos was ~460-fold greater at 60 °C compared to 15 °C after 8 h. All temperatures greater than 35 °C, for concentrate, resulted in significantly greater permeation (at 8 h) (*p* < 0.0001) with each increase in temperature, whereas for application strength, exposure significantly greater permeation was only seen for the highest tested temperature (i.e., 60 °C statistically different to all other temperatures; *p* = 0.0001). Maximum permeation rates over the 8 h exposure timeframe, along with breakthrough time, defined as the point at which the permeation rate reaches 1 µg/cm^2^/min, are presented in Table 1. As the temperature was increased, a substantial increase in maximum permeation rate through PVC gloves was observed for both concentrate and application strength dichlorvos. For example, concentrate permeation rates were ~160-fold greater at 60 °C versus 15 °C. A steady decrease in breakthrough time was also observed for concentrate exposure (>8 h at 15 °C to 2 h at 60 °C). In contrast, for application strength exposure, a breakthrough time below 8 h was only observed at 60 °C (3 h). Table 2 summarizes data obtained from Arrhenius plots, where good linear correlations (R2 ≥ 0.88) were obtained between K_p_ and 1/T for concentrate dichlorvos. Changes in *E_a_* (15−45 °C) and *E_a_* (25−60 °C) are reported and show a greater than 2-fold reduction in activation energy at the elevated temperature range (25−60 °C).

### 3.2. Skin Permeation Studies

The cumulative transdermal penetration of concentrate dichlorvos (1398 g/L) at 8 h was 179 ± 74 µg/cm^2^ and 1315 ± 362 µg/cm^2^ at 25 and 37 °C, respectively, whereas the cumulative penetration for application strength (6 g/L) was 29.8 ± 5.7 µg/cm^2^ and 115 ± 19 µg/cm^2^ at 25 and 37 °C, respectively. This corresponds to a significantly greater permeation of dichlorvos after 8 h, for both concentrate and application strength, at 37 °C compared to 25 °C (concentrate; *p* = 0.0032; application strength: *p* = 0.0131). The calculated steady-state fluxes (*J_ss_*), permeation constants (*K_p_*), and lag times are shown in Table 3. Good correlation of the linear equations used to calculate steady state flux’s were achieved (R^2^ > 0.98). An increase in both *J_ss_* and *K_p_* was observed upon increasing the temperature from 25 to 37 °C for concentrate dichlorvos. This observation aligned with a corresponding decrease in lag time. Steady-state was not achieved within the experimental timeframe for application strength dichlorvos at 25 °C, preventing an empirical comparison of flux values due to temperature effects at this exposure concentration.

## 4. Discussion

This is the first study evaluating the influence of elevated temperatures on the barrier effectiveness of both non-disposable PVC gloves and human skin for dichlorvos. The performance of chemical protective gloves worn during handling of pesticides in agricultural environments is a critical factor, as they are often employed as the frontline control measure to prevent dermal uptake. Our research has highlighted the importance of elevated temperature conditions on glove protection performance and dermal risks.

Two exposure concentrations were chosen to represent those commonly handled during OP preparation/mixing and application/spraying in agricultural settings. Non-disposable PVC gloves were studied as these are the commonly recommended protection, and variable temperature experiments were undertaken to understand their effectiveness in hot environments commonly found in outdoor agriculture. When exposed to concentrated dichlorvos, PVC gloves afforded protection against chemical breakthrough ranging from 2 h at 60 °C up to greater than 8 h at 15 °C. In comparison, when exposed to application strength dichlorvos, the chemical breakthrough was only observed within the 8 h experimental timeframe at 45 and 60 °C, An approximate 160-fold increase in maximum permeation rate was observed at 60 °C compared to 15 °C for both concentrate and application strength exposures. The maximum permeation rate recorded for application strength was only 2.2% of the maximum rate recorded for concentrate. A previous study investigating dichlorvos permeation through PPE material recorded no breakthrough through rubberized fabric (0.42 mm) for a 0.1 mg/mL dichlorvos solution within an 8 h experimental timeframe. This is consistent with the data obtained in this study for application strength (6 g/L) at low temperatures. A further preliminary study of dichlorvos permeation through disposable nitrile and neoprene gloves has been previously undertaken [31]. For both nitrile and neoprene gloves, the breakthrough times for 4 h periods for concentrate at 23 and 45 °C were <5 min. For application strength at 23 and 45 °C they were >4 h and 40 min. However, double gloving (nitrile/neoprene) increased breakthrough time for 4 h periods for concentrate at 23 and 45 °C to 30 min and 20 min, respectively. For application strength at 23 and 45 °C, they were >4 h and 180 min, respectively. This markedly improved performance was attributed to the combined polar and non-polar surfaces [32].

Activation energy (*E_a_*) provides a measure of the resistance to diffusion of a penetrant to cross a given membrane and generally, the value of the activation energy is a function of both the diffusing molecule and the diffusion pathway [33]. The activation energy of the PVC gloves was shown to decrease more than 2-fold at an elevated temperature range (25–60 °C) when compared to a lower temperature range (15–45 °C). At a fundamental level, this indicates a reduced diffusion resistance for dichlorvos at elevated temperatures that may be due to the relaxing of the PVC material, thus allowing greater chemical penetration through micropores in the polymer structure [34]. These observations are consistent with the reduced chemical resistive properties of the glove material, as evidenced by the decreased breakthrough times and increased maximum permeation, at higher temperatures.

One of the problems associated with non-disposable gloves is the potential for internal contamination and subsequent ongoing exposure following re-use [35]. The use of disposable gloves obviates this problem. Indeed, it has been argued that the use of disposable gloves may be preferable, as workers may have a false sense of security with thicker gloves [36]. Our data suggest that non-disposable PVC gloves provide good protection assuming no internal contamination. Nevertheless, double gloving (nitrile and neoprene) is a very viable alternative (breakthrough time is at least 20 min even with concentrate at 45 °C).

Skin permeation of dichlorvos was evaluated in vitro under infinite dose conditions using human epidermis. When applied at concentrate (1398 g/L) and application (6 g/L) concentrations, dichlorvos displayed a propensity to permeate through human skin. Increasing temperature from 25 to 37 °C resulted in an approximate 13-fold and 4-fold increase in cumulative skin permeation for concentrate and application concentrations, respectively. IH SkinPerm (v2.05) is a mathematical model used to estimate transdermal permeation of chemicals in solution [37]. The 8 h cumulative permeation results at 25 °C obtained in this study (concentrate: 179 ± 37 µg/cm^2^; application strength: 29.8 ± 5.7 µg/cm^2^) compare remarkably well with those predicted by the IH SkinPerm model (concentrate: 116 µg/cm^2^; application strength: 35 µg/cm^2^). The steady state flux (*J_ss_*) and permeation constant increased approximately 12-fold for concentrate dichlorvos, upon increasing the temperature to 37 °C. Moore et al. [16] reported steady state flux values for dichlorvos, in a variety of different vehicles, applied to human skin in vitro under both finite and infinite dose conditions. Their flux values for infinite dose application of 10 mg/mL dichlorvos in three different vehicles ranged from 108 to 1346 µg/cm^2^/h [16]. Our calculated flux value for application strength at 37 °C was 15.0 ± 0.59 µg/cm^2^/h. Despite being lower, this value compares favorably to those presented by Moore et al., given dichlorvos was delivered to the skin in vehicles known to enhance dermal penetration in that study [16]. The larger flux values recorded for concentrate dichlorvos in this study provides empirical evidence to confirm its ability to permeate human skin, in large quantities, in the absence of a vehicle or co-solvent. These outcomes support the conclusion of Moore et al. that dichlorvos poses a significant dermal exposure risk under infinite and finite dose conditions [16]. Further, this study also supports the observations of Perger [15] that total body dermal exposure to high concentrations of dichlorvos presents a significant toxicity risk, particularly at elevated temperatures.

The results presented in this study have a high degree of occupational relevance for the agriculture industry and can assist in informing necessary exposure reduction measures. The skin penetration data highlight the need for control measures and personal hygiene practice (i.e., regular hand washing) to prevent potential exposure to dichlorvos during handing tasks. The data also demonstrate the significant influence that in-use temperature conditions have on protective glove performance and the potential uptake of dichlorvos. Glove flexing and abrasion likely encountered during in-use conditions could further contribute to reduced protection performance, but this was not tested in the current study. Whilst only in-use conditions were considered in this study, the storage conditions of gloves prior to use is an area that warrants further investigation in the future. Exposure of gloves to UV and elevated temperatures is common during storage in agricultural contexts and it is not known what effect this may have on their ultimate protection performance.

The findings have a number of practical implications for the recommended usage conditions of protective gloves in agricultural contexts involving hot environments. Thus, it may be warranted to recommend a regular glove replacement strategy, with more frequency in hot conditions. Glove manufacturers may also wish to consider testing glove performance outside standard conditions to better encompass a range of in-use conditions in order to provide further recommendations to end-users.

## 5. Conclusions

In conclusion, this work has shown that elevated temperature conditions have the potential to significantly influence protective glove performance and subsequently dermal absorption risk of occupational relevance for the use and handling of dichlorvos. Under realistic skin exposure scenarios, dichlorvos readily penetrated bare skin, reinforcing the need for adequate protective gloves. The recommended PVC gloves tested in this study appear to provide good protection against dichlorvos permeation under typical handling activities and conditions (e.g., spraying dilute concentrations and moderate ambient temperatures). However reduced performance may occur under hot environmental conditions, especially for tasks involving mixing/handling of the concentrated product. As such, it is important to consider in-use conditions including high temperature environments when implementing best practice for glove use and replacement strategies.

## Figures and Tables

**Table 1:** Breakthrough times and maximum permeation rates for performance testing of elbow length PVC gloves at varying temperatures. Values are reported as mean ± SEM (n ≥ 3).

	Temperature (°C)	Breakthrough Time ^1^ (hours)	Maximum Permeation Rate (µg/cm^2^/min)
**Concentrate**	15	>8	0.86 (±0.33)
	25	4	14.1 (±1.1)
	35	4	22.4 (±3.5)
	45	3	102 (±0.3)
	60	2	137 (±60)
**Application Strength**	15	>8	<LOD
	25	>8	0.0188 (±0.005)
	35	>8	0.196 (±0.03)
	40	>8	0.476 (±0.13)
	45	8	1.73 (±0.38)
	60	3	3.02 (±1.0)

^1^ Defined as the time at which the permeation rate reaches 1 µg/cm^2^/min. LOD = 0.14 µg/mL.

**Table 2:** Activation energy (E_a_) and correlation coefficients (R^2^) of the PVC glove membrane over different temperature ranges for concentrate dichlorvos.

	Δ*E_a_* 15–45 °C (kJ/mol)	Δ*E_a_* 25–60 °C (kJ/mol)
**Concentrate**	45.0 (R^2^ = 0.88)	20.1 (R^2^ = 0.92)

**Table 3:** Skin permeation of dichlorvos through human epidermis at different dichlorvos concentrations and temperatures. Values are presented as mean ± SEM (n ≥ 4).

	Temperature (°C)	*J*_ss_ (µg/cm^2^/h)	K_p_ (10^−4^ cm/h)	Lag Time (h)
**Concentrate**	25	30.4 (±1.1)	0.217 (±0.008)	2.2
	37	277 (±0.03)	1.52 (±0.0002)	1.8
**Application Strength**	25	ND	ND	ND
	37	15.0 (±0.6)	25.0 (±0.98)	0.38

ND = Not determined.
